# Genetic diversity of vector-borne pathogens in ixodid ticks infesting dogs from Pakistan with notes on *Ehrlichia canis*, *Rickettsia raoultii* and *Dirofilaria immitis* detection

**DOI:** 10.1186/s13071-023-05804-2

**Published:** 2023-06-28

**Authors:** Jehan Zeb, Baolin Song, Munsif Ali Khan, Haytham Senbill, Muhammad Umair Aziz, Sabir Hussain, Abdul Waris, Ala E-Tabor, Olivier Andre Sparagano

**Affiliations:** 1 Department of Infectious Diseases and Public Health, Jockey Club College of Veterinary Sciences, City University of Hong Kong, Kowloon, 518057, 999077 Hong Kong, SAR China; 2 Vector-Borne Diseases Control Unit, District Health Office, Abbottabad, 22010 Pakistan; 3 Department of Applied Entomology and Zoology, Faculty of Agriculture, Alexandria University, Alexandria, 21545 Egypt; 4 Department of Biomedical Sciences, City University of Hong Kong, Kowloon, 518057, 999077 Hong Kong, SAR China; 5 Queensland Alliance for Agriculture and Food Innovation, The University of Queensland, St Lucia, QLD Australia

**Keywords:** *Ehrlichia canis*, *Rickettsia raoultii*, *Dirofilaria immitis*, *Wolbachia* species, Tick, Dog, Pakistan

## Abstract

**Background:**

Vector-/tick-borne pathogens (V/TBPs) pose a potential threat to human and animal health globally. Information regarding canine V/TBPs is scarce and no specific study has been conducted so far to explore the microbial diversity within ticks infesting dogs from Pakistan. Herein, this knowledge gap is addressed by assessing the genetic diversity and prevalence pattern of V/TBPs in ixodid ticks with special implications for public and canine health.

**Methods:**

A total of 1150 hard ticks were collected from 300 dogs across central Khyber Pakhtunkhwa (KP), Pakistan. After morpho-molecular identification, 120 tick samples were screened for the presence of V/TBPs by amplifying *16S rRNA*/*gltA* (*Rickettsia*/*Ehrlichia* and *Wolbachia* sp.), *18S rRNA* (*Theileria* sp.) and *cox1* (*Dirofilaria* sp.) genes through PCR followed by sequencing and phylogenetic study.

**Results:**

In toto, 50 ixodid ticks (50/120, 41.7%) were found positive for V/TBPs DNA. The detected V/TBPs were categorized into five genera and eight species, viz. *Ehrlichia* (*E. canis* and *Ehrlichia* sp.), *Rickettsia* (*R. massiliae, R. raoultii* and *Rickettsia* sp.), *Theileria* (*T. annulata*), *Dirofilaria* (*D. immitis*) and *Wolbachia* (*Wolbachia* sp.). The pathogen prevalence patterns showed that *R. massiliae* was the most prevalent zoonotic V/TBP (19.5%), followed by *E. canis* (10.8%),* Rickettsia* sp. (7.5%)*, R. raoultii* (6.7%), *T. annulata* (5.8%), *D. immitis* (5.8%), *Wolbachia* sp. (4.2%) and *Ehrlichia* sp. (3.3%), respectively. Among the screened tick species, most *Rhipicephalus sanguineus* sensu lato samples were found positive for V/TBP DNA (20/20,100%) followed by *Rh. turanicus* sensu stricto (13/20, 65%), *Hyalomma dromedarii* (8/20, 40%), *Rh. haemaphysaloides* (6/20, 30%), *Hy. excavatum* (2/20, 10%) and *Rh. microplus* (1/20, 5%). Co-occurrence of V/TBP was also detected in tick specimens (single V/TBP infection: 32 ticks; double and triple: 13 and 5 tick samples). The detected pathogens shared a phylogenetic relationship with similar isolates published in NCBI GenBank from Old and New World countries.

**Conclusion:**

Ixodid ticks infesting dogs harbor a diverse array of V/TBPs including zoonotic agents from Pakistan. Furthermore, the presence of *D. immitis* in ticks that infest dogs raises the possibility that this parasite has either attained its dead-end host (i.e. the tick) while feeding on dogs or has expanded its range of intermediate/paratenic hosts. Further research work is needed to investigate the epidemiology and confirm the vector competence of screened tick species for these pathogens from Pakistan.

**Graphical Abstract:**

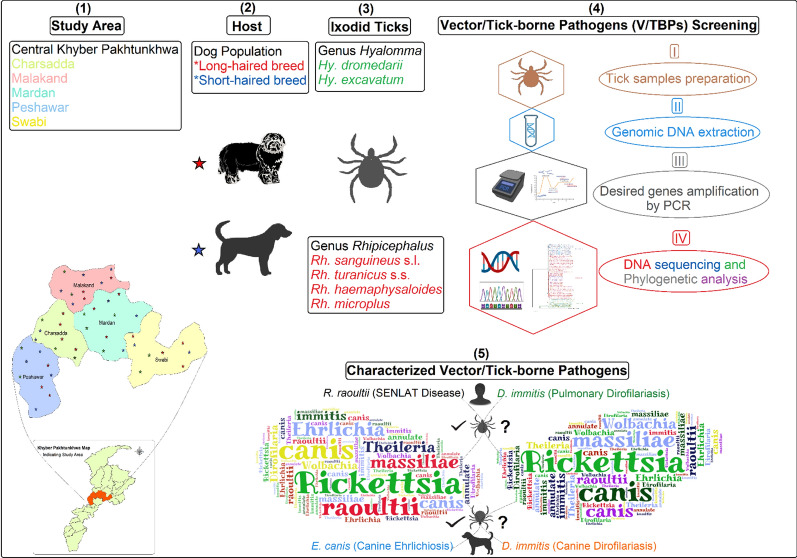

**Supplementary Information:**

The online version contains supplementary material available at 10.1186/s13071-023-05804-2.

## Background

Ticks are hematophagous arthropods infesting livestock/companion animals including dogs and are responsible for transmitting life-threatening pathogens infecting humans and animals worldwide [1, 2]. Research on tick and tick-borne diseases/pathogens (TBPs) has received considerable attention in the recent era of changing climatic scenarios which have resulted in global average temperature rise and annual rainfall shift, thus creating suitable biotopes in different landscapes for the survival and development of disease vector species. Consequently, the zoogeographical range of disease vectors and their associated pathogens expands, putting the public and veterinary health workers at risk of contracting V/TBPs infection [3, 4]. Globally ticks are known as vectors and reservoirs for several pathogenic microbes, i.e. viruses, bacteria (including *Rickettsia*) and protozoans [5]. Ticks stand second to the mosquito as a potent vector for transmitting infectious diseases to humans and animals [6, 7]. Ixodid tick attachment to the host and prolonged hematophagy may facilitate the transfer of TBPs from an infected tick to the animal hosts which in turn ensures pathogen dispersal to other geographic localities through pet/livestock intercontinental transportation [8].

Among TBPs, ehrlichiosis (canine monocytic ehrlichiosis/tropical canine pancytopenia) adversely affects dog health. Its etiological agent, *E. canis*, is an obligate intracellular alpha‐proteobacterium (of the Rickettsiae group), which replicates within mononuclear cells of the host [9]. There have been reports of canine ehrlichiosis from subtropical and tropical regions of the world [10]. *Ehrlichia* species can also infect humans and several other animal species like cats and horses. Important species of the genus *Ehrlichia* are *E. chaffeensis*, *E. canis*, *E. ewingii*, *E. muris*, *E. sennetsu*, *E. risticii*, *E. equi* and *E. ruminantium* [11]. *Ehrlichia canis* is among the TBPs that can infect dogs [12]. Infected dogs present a wider spectrum of ehrlichiosis consisting of three stages: acute, subclinical and clinical or chronic infection characterized by pyrexia, lymphadenitis, respiratory distress, body weight loss, bleeding episodes (epistaxis), visual and neurological involvement (blindness and meningitis) [13]. Dogs, red foxes and yellow jackals serve as probable reservoir hosts of *E. canis*, while the brown dog tick, *Rhipicephalus sanguineus* s.l., is the major arthropod vector of *E. canis* [14]. *Rhipicephalus sanguineus* s.l. ticks have a broader zoogeographical range that typically infests canids (dogs) and is most frequently found on dogs in South Asia including Pakistan [15]. *Ehrlichia canis* has zoonotic importance and is horizontally transmitted to humans by these ticks [16–18].

The rickettsial bugs constitute an important group of TBPs responsible for a wide spectrum of infections in humans and animals from subclinical to severe life-threatening forms [19–21]. Rickettsial pathogens have a cosmopolitan distribution and are grouped into two classes infecting humans and animals including dogs: (i) the typhus group (TG: *Rickettsia prowazekii* and *R. typhii*) and (ii) the spotted fever group (SFG: *R. rickettsii*, *R. slovaca*, *R. sibirica*, *R. raoultii*, *R. conorii*, *R. peacockii*, *R. honei*, *R. japonica*, *R. montanensis*, *R. massiliae*, *R. ripicephali*, *R. amblyommii*, *R. africae*, *R. parkeri*, *R. heilongjiangensis* and *R. phillipi*). The typhus and SFG rickettsiae are vectored by ixodid ticks, lice, fleas and other blood-sucking arthropods [22–24].

The SFG rickettsial microbe *R. raoultii* is the etiological agent of human scalp eschar and neck lymphadenopathy (SENLAT) across the globe. *Rickettsia raoultii* was first detected in *Dermacentor nuttalli* and *Rhipicephalus pumilio* ticks (as novel rickettsial genotypes: DnS14, DnS28 and RpA4) from the Siberian region of Russia at the end of the twentieth century [25]. The principal arthropod vectors involved in its transmission are *Dermacentor* ticks. However other ixodid ticks of the genera *Amblyomma, Haemaphysalis, Hyalomma* and *Rhipicephalus* were also incriminated in *R. raoultii* transmission to humans. To date, *R. raoultii*'s presence has been confirmed in many Asian and European countries in ixodid ticks, except Pakistan [26].

The first human infection with *R. raoultii* was reported in a Spanish resident around 2006 [27]. The clinical manifestations of human SENLAT are a history of a tick bite, skin lesion at the site of tick bite/inoculation eschar on the scalp (because *Dermacentor* ticks prefer hairy prey), draining neck lymph node inflammation and surrounding alopecia, rarely with fever and rashes [26, 28].

*Dirofilaria immitis*, commonly known as dog heartworm, is a nematode parasite that causes fatal illness in dogs, known as cardiopulmonary dirofilariasis or dog heartworm disease [29, 30]. *Dirofilaria* species can also infect other animals including cats, badgers, coyotes, jackals, wolves and horses [29]. However, *D. immitis*, *D. repens*, *D. tenuis, D. striata, D. ursi* and *D. subdermata* have anthropozoonotic potential and have been detected in human patients. Human heartworm infection is relatively uncommon and categorized as pulmonary dirofilariasis (caused by *D. immitis*) and subcutaneous dirofilariasis (caused by *D. repens, D. tenuis* and other associated species) [31]. Canine dirofilariasis is prevalent in tropical, subtropical and temperate regions of the world [32]. Dirofilarial nematodes are vectored by the hematophagous female culicid mosquito of the genera *Aedes, Culex* and *Anopheles* [29, 32]. Canine dirofilariasis in dogs involve respiratory and cardiac complications with a typical clinical picture of chronic non-productive cough, shortness of breath, reduction in body weight, asthenia, epistaxis, cyanosis and congestive heart failure [30].

*Wolbachia* is an endosymbiotic *α*-proteobacteria (Rickettsiales) that shares a close genome resemblance with *Anaplasma* and *Ehrlichia* species [33]. These endosymbionts are widely distributed and detected in almost all arthropods and other invertebrates including filarial nematodes. Five decades earlier, the first bacteria-like microorganism was detected in the filarial worm *D. immitis*. Later, molecular findings showed that these bacterial bugs belong to the genus *Wolbachia* [34]. *Wolbachia* sp. exists in symbiotic associations (mutualism and parasitism) with roundworm microfilariae and influences its reproductive biology [35]. Research advancement with a focus on the interaction between *Wolbachia* and *D. immitis* has shown that these *Wolbachia* play a vital role in the development (embryogenesis and molting) of nematode microfilariae [36] while growing microfilariae provide essential nutrients (amino acids) for *Wolbachia* survival in a mutualistic manner [37]. The *Wolbachia* strains prevalent across diverse host species are categorized into different subgroups (each subgroup constitutes a separate phylogenetic lineage). In the beginning, *Wolbachia* endosymbionts were placed in subgroups A and B (infecting arthropods) followed by the addition of two more subgroups, C and D, after *Wolbachia* strains were discovered in parasitic nematode filariae. The ongoing discovery of the *Wolbachia* strain has resulted in *Wolbachia* subgroup multiplication and further creation of ten subclades, E, G, H, I, K, M, N, O, P and Q, infecting arthropods, one subclade J infecting filariae, one subclade L discovered in the plant-associated parasitic nematode (*Radopholus similis*) and one subgroup F infecting both arthropods and filariae [33, 38].

Dogs have been domesticated by humans since prehistoric times and live as pets in close association with humans [15]. Owing to massive population growth and global pet mobility, dogs are prone to contracting fatal parasitic illnesses including vector-borne diseases at the same time or in succession to locations where canine vector-borne diseases are endemic [8, 39]. Dogs also serve as preferred hosts of numerous hard tick species harboring zoonotic pathogens, thus increasing the likelihood of human infestation by these ticks and the eventual transfer of TBPs [40]. To track the TBPs of canids in a particular geographic location, the availability of blood-sucking arthropods transmitting these TBPs is a reliable marker for tracking pathogens in that particular region [25].

Currently, there is no comprehensive scientific report available on V/TBPs circulating in the tick vector and dog populations from Pakistan with implications for public and canine health. To address this crucial knowledge gap and provide baseline support for designing effective vector control strategies in the future as well as to educate dog owners about the risk of contracting V/TBPs infection, this scientific work explored the genetic diversity of pathogens in ixodid ticks collected from dogs.

## Methods

### Ethical consideration

This study was approved by the ethics committee on animal care and use in scientific experimentations at the College of Veterinary Sciences and Animal Husbandry, Abdul Wali Khan University Mardan, Pakistan (CVSAH/FCLS/AWKUM/2021/228).

### Study location and tick sampling

**Fig. 1:**
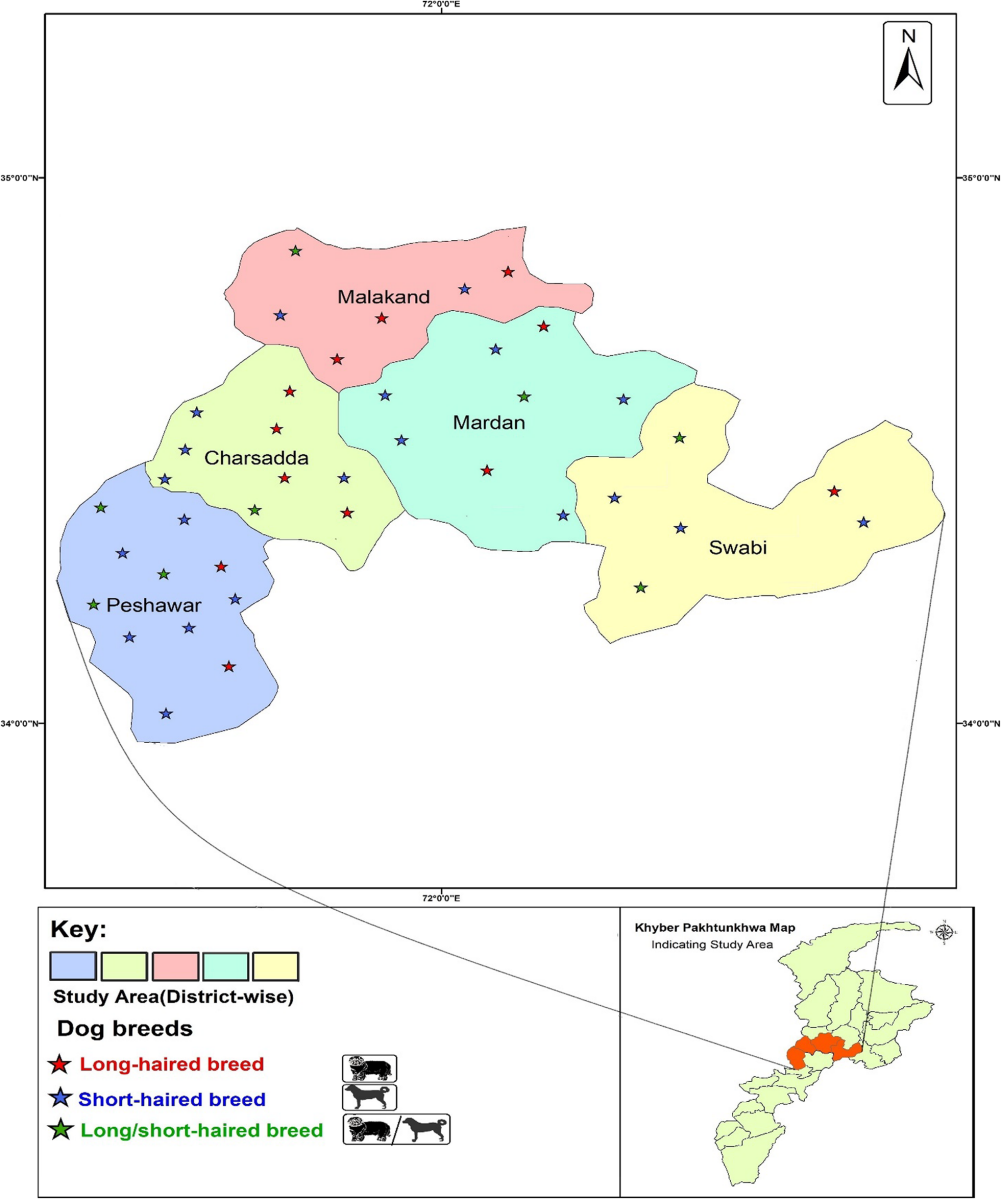
Map of study area districts located in central KP, Pakistan (where ixodid ticks were sampled from dogs)

A total of 1150 ixodid ticks were collected from 300 pet dogs between January 1, 2022, and August 30, 2022. The dog population (*n* = 300, short-haired breed = 182, long-haired breed = 118) was examined for tick infestation and collection in 40 different towns of 5 districts located centrally in KP, Pakistan (Fig. 1). The collected ticks were stored in 70% ethanol and shipped to the public health laboratory at the City University of Hong Kong for further investigations. A comprehensive report has been published elsewhere that provides details about the present study area, its climatic conditions, dominant vegetation type, study design, tick sampling strategy as well as host demography [15].

### Tick morphological identification and DNA extraction

The collected ticks were morphologically identified up to species level under a stereo zoom microscope (Olympus^®^, Tokyo, Japan) using standard morpho-taxonomic keys [41, 42] followed by molecular confirmation. The ixodid fauna was categorized into six species of hard ticks, viz. *Rhipicephalus sanguineus* s.l., *Rh. turanicus* s.s., *Rh. haemaphysaloides*, *Rh. microplus*, *Hyalomma dromedarii* and *Hy. excavatum*. Among the identified ticks, 120 hard ticks (20 specimens of each tick species) were selected for DNA isolation and pathogen screening. Genomic DNA was extracted from tick samples individually using a DNA extraction kit (QIAamp^®^ DNA Mini Kit, Qiagen, Hilden, Germany) following the manufacturer's DNA extraction protocols. The DNA in the samples was analyzed qualitatively and quantitatively using a spectrophotometer (NanoDrop™ Thermo Scientific™, Waltham, MA, USA). All the DNA samples were preserved in a deep freezer at − 80 °C till further analysis.

### Molecular screening of V/TBPs through PCR

For pathogen detection in ixodid tick species, three sets of PCRs were performed, a first PCR for the detection of *Anaplasma*/*Ehrlichia*/*Rickettsia* species using *16S rRNA*/*gltA* gene primers sets, a second PCR for the detection of *Theileria*/*Babesia* species using *18S rRNA* gene primer set and a third PCR for the detection of *Dirofilaria* species using *cox1* gene primer set (Table 1). All PCR reactions were performed in a total reaction volume of 35 µl containing 17.5 µl Master Mix, 3 µl genomic DNA, 1 µl of each primer (forward and reverse, 10 pmol) and 12.5 µl PCR-grade water. The PCR conditions for *Anaplasma*/*Ehrlichia*/*Rickettsia 16S rRNA*/*gltA* were: initial denaturation of template DNA samples at 95 °C for 5 min followed by 35 cycles of denaturation at 95 °C for 30 s, annealing at 60 °C/55 °C (*16S rRNA*/*gltA*) for 30 s, extension at 72 °C for 30 s and a final extension at 72 °C for 10 min [43–45]. The thermodynamic conditions set in a thermocycler for Piroplasms *18S rRNA* amplification were: an initial denaturation at 95 °C for 3 min followed by 35 cycles of denaturation at 94 °C for 1 min, annealing at 54 °C for 30 s, extension at 72 °C for 30 s and final extension at 72 °C for 10 min [46]. The *Dirofilaria cox1* gene was amplified with the following thermocycling parameters: an initial denaturation at 95 °C for 3 min followed by 35 cycles of denaturation at 95 °C for 1 min, annealing at 40 °C for 1 min, extension at 72 °C for 1.5 min and final extension at 72 °C for 7 min [47]. The amplified PCR products were confirmed by submarine gel electrophoresis using 2% agarose gels stained with ethidium bromide and visualized under UV radiation in a Gel-Doc machine (Bio-Rad Laboratories, Hercules, CA, USA).

**Table 1:** List of primer sets used for the detection/amplification of V/TBP genes in ixodid ticks

Desired organism	Target gene	Primer	Primer sequence (5′–3′)	Amplicon size (bp)	References
*Anaplasma/Ehrlichia* species	*16S rRNA*	EHR16SF	GGT ACC YAC AGA AGA AGT CC	~ 345	[43]
		EHR16SR	TAG CAC TCA TCG TTT ACA GC		
*Rickettsia* species	*16S rRNA*	Rick-F1	GAA CGC TAT CGG TAT GCT TAA CAC A	~ 364	[44]
		Rick-R2	CAT CAC TCA CTC GGT ATT GCT GGA		
	*gltA*	CS-78	GCA AGT ATC GGT GAG GAT GTA AT	~ 401	[45]
		CS-238	GCT TCC TAA AAT TCA ATA AAT CAG GAT		
*Babesia/Theileria* species	*18S rRNA*	NWF	GTC TTG TAA TTG GAA TGA TGG	~ 500	[46]
		NWR	TAG TTT ATG GTT AGG ACT ACG		
*Dirofilaria* species	*cox1*	LCOI490	GGT CAA CAA ATC ATA AAG ATA TTG	~ 710	[47]
		HCO2198	TAA ACT TCA GGG TGA CCA AAA AAT CA		

*16S rRNA:* 16S ribosomal RNA gene; *18S rRNA:* 18S ribosomal RNA gene; *gltA:* Rickettsial citrate synthase-encoding gene; *cox1*: Cytochrome c oxidases subunit 1 gene

### PCR product purification and sequencing

All the PCR products of desired genes were sent to a genome sequencing facility (BGI Tech Solutions, Hong Kong Co. Ltd., SAR China) for purification and unidirectional DNA sequencing. The query sequence dataset of V/TBPs was edited for trimming unnecessary nucleotides at termini and aligned in MEGA7 [48]. An online BLAST analysis of the query dataset was performed on the NCBI GenBank server to identify and characterize the sequenced pathogens. The reference sequences with query coverage of 99%–100% were downloaded as separate datasets for phylogenetic analysis of sequenced microorganisms. Finally, the *16S rRNA*, *gltA*, *18S rRNA* and partial *cox1* nucleotide sequences of V/TBPs were submitted to the NCBI GenBank Nucleotide repository, and accession numbers were assigned.

### Phylogenetic analyses of V/TBPs

Molecular phylogenies of V/TBPs were inferred from the partial nucleotide sequences of amplified genes using MEGA-7 software [48]. The maximum likelihood (ML) algorithm-based phylogenetic trees were constructed for *Rickettsia*, *Ehrlichia,* and *Wolbachia* sp. using *16S rRNA* nucleotide sequences. Additionally, rickettsial isolates were further characterized by using partial *gltA* nucleotide sequence-based phylogram. Similarly, the same algorithm (ML) was applied to infer the evolutionary histories of *Theileria* and *Dirofilaria* isolates by using their target genes' partial nucleotide sequences (*18S rRNA* and *cox1* genes). The whole dataset was resampled 1000 times for bootstrap value generation with Kimura two-parameter model for *16S rRNA*- and *18S rRNA*-based phylogenetic trees and Tamura three-parameter model for rickettsial *16S rRNA*, *gltA* and dirofilarial *cox1* isolates. These best-fit models of sequence evolution/phylogram construction were chosen based on the lowest Bayesian information criterion scores (BIC), corrected Akaike information criterion value (AIC) and maximum likelihood value (*Inl*) [49].

### Statistical analyses

The dataset generated from this research work was analyzed statistically. The prevalence/infection rate of detected pathogens was calculated as the number of infected ticks (individual tick species) by V/TBP species divided by the total number of molecularly screened tick specimens (*n* = 120). Differences in the prevalence of pathogens in various tick species were assessed by Chi-square statistic in GraphPad software (Version 9.1.0, Boston, MA, USA). The significance level was set at *P* < 0.05.

## Results

### Detection, diversity and prevalence of V/TBPs in ixodid ticks

Out of 120 molecularly screened ixodid ticks, V/TBPs were detected in 50 (41.7%) tick specimens. The characterized pathogens were categorized into five genera and eight species, viz. *Ehrlichia* (*E. canis,* and *Ehrlichia* sp.); *Rickettsia* (*R. massiliae*, *R. raoultii*, and *Rickettsia* sp.); *Theileria* (*T. annulata*); *Dirofilaria* (*D. immitis*) and *Wolbachia* (*Wolbachia* sp.). Genus-based analysis showed that Rickettsial pathogens were most common (33.7%) followed by Ehrlichial microorganisms (14.1%), *Theileria* (5.8%), parasitic *Dirofilaria* of a dog (5.8%) and *Wolbachia* (4.2%). The species-wise prevalence pattern showed that *R. massiliae* was the most prevalent zoonotic V/TBP (19.5%), followed by *E. canis* (10.8%)*, Rickettsia* sp. (7.5%), *R. raoultii* (6.7%), *T. annulata* (5.8%), *D. immitis* (5.8%), *Wolbachia* sp. (4.2%) and *Ehrlichia* sp. (3.3%), respectively (Table 2).

**Table 2:** Molecular detection of V/TBPs in hard tick species collected from dogs across central Khyber Pakhtunkhwa, Pakistan

Study area (districts)	Ixodid tick species	Canine associated vector/tick-borne pathogens								Ticks screened *n*/*t* (%)
		Genus *Ehrlichia*		Genus *Rickettsia*			Genus *Theileria*	Genus *Dirofilaria*	Genus *Wolbachia*	
		*E. canis*	*Ehrlichia* sp.	*R. massiliae*	*R. raoultii*	*Rickettsia* sp.	*T. annulata*	*D. immitis*	*Wolbachia* sp.	
Charsadda	*Rh. sanguineus* s.l.	+	+	+	+	+	−	+	+	20/20 (100%)
Malakand	*Rh. turanicus* s.s.	+	+	+	+	+	−	+	+	13/20 (65%)
Mardan	*Rh. haemaphysaloides*	+	+	+	−	+	+	−	−	6/20 (30%)
Peshawar	*Rh. microplus*	−	−	+	−	−	−	−	−	1/20 (5%)
Swabi	*Hy. dromedarii*	−	−	+	−	+	+	−	−	8/20 (40%)
	*Hy. excavatum*	−	−	+	−	−	+	−	−	2/20 (10%)
Total n (%)	V/TBP Sp	13 (10.8%)	4 (3.3%)	23 (19.5%)	8 (6.7%)	9 (7.5%)	7 (5.8%)	7 (5.8%)	5 (4.2%)	
	V/TBP G	17 (14.1%)		40 (33.7%)			7 (5.8%)	7 (5.8%)	5 (4.2%)	50/120 (41.7%)

*n*/*t* (%): *n*: number of positive ticks, *t*: total ticks screened, %: percent positive tick; *n* (%): *n*: number of positive tick species, %: pathogen/endosymbiont prevalence in all tick species; V/TBP Sp./G.: vector/tick-borne pathogen species/genus prevalence; +/−: positive/negative

**Fig. 2:**
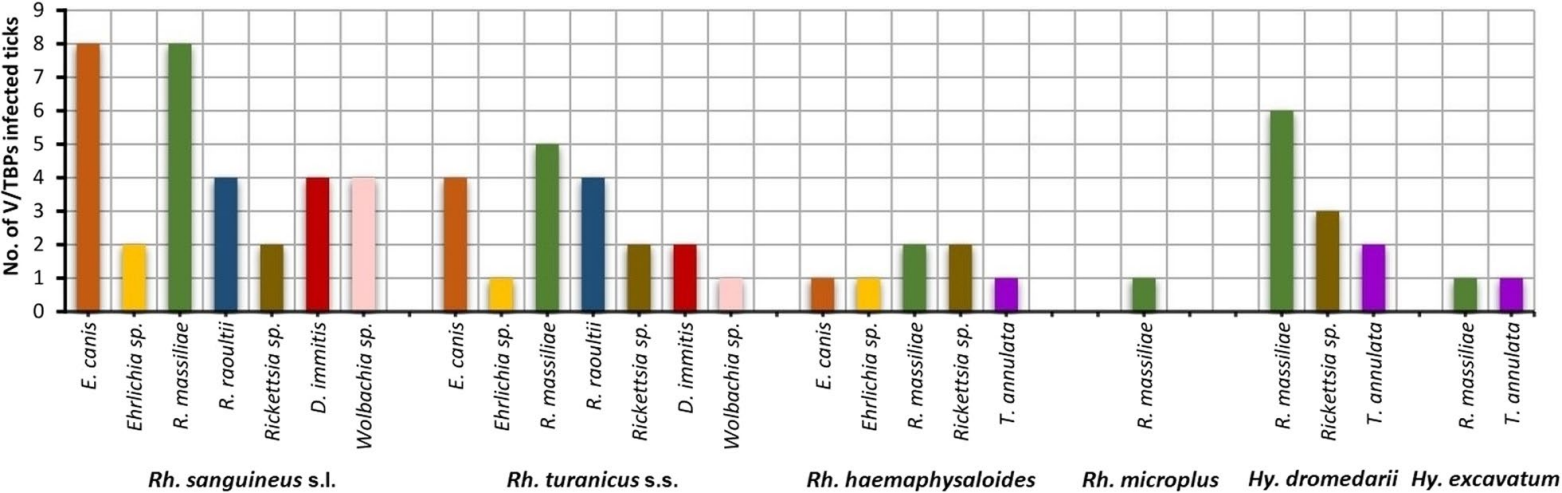
Vector/tick-borne pathogen species detected in each ixodid tick species collected from dogs across the study area

Among the molecularly screened hard tick species for pathogen detection, *Rh. sanguineus* s.l. was the most infected tick species by V/TBPs (20/20, 100%) followed by *Rh. turanicus* s.s. (13/20, 65%), *Hy. dromedarii* (8/20, 40%), *Rh. haemaphysaloides* (6/20, 30%), *Hy. excavatum* (2/20, 10%) and *Rh. microplus* (1/20, 5%). The occurrence of V/TBPs in ticks exhibits significant variations in different tick species (*χ*^2^ = 11.998, *df* = 5, *P* = 0.004). Rich pathogens diversity (number of species) was reported in *Rh. sanguineus* s.l. and *Rh. turanicus* s.s. (7/8 V/TBP species) followed by *Rh. haemaphysaloides* (5/8), *Hy. dromedarii* (3/8), *Hy. excavatum* (2/8) and *Rh. microplus* (1/8) (Table 2) (Fig. 2).

### Single and multiple V/TBP infection in ixodid ticks

Single and mixed infections (co-infection: the presence of multiple V/TBP species within individual ticks) were observed within screened tick species. Out of 50 pathogen-positive ticks, 32 (64%) were found positive for a single V/TBP infection. Single pathogen prevalence pattern showed that *R. massiliae* was a highly prevalent zoonotic pathogen (22%) in ixodid ticks followed by *R. raoultii* (8%), *Rickettsia* sp. (8%), *E. canis* (6%), *Ehrlichia* sp. (6%), *T. annulata* (6%), *D. immitis* (2%) and *Wolbachia* sp. (2%). However, mixed/co-infection patterns revealed that 13 (26%) ticks were infected with two V/TBPs. Double co-infection in hard ticks showed that the most prevalent V/TBPs, i.e. *R. massiliae* + *Rickettsia* sp. (8%) detected in *Rh. sanguineus* s.l., *Rh. turanicus* s.s., *Rh. haemaphysaloides* and *Hy. dromedarii* ticks followed by *E. canis* + *R. massiliae* (6%) double coinfection in *Rh. sanguineus* s.l. and *Rh. turanicus* s.s.; *R. massiliae* + *R. raoultii* (4%) double co-infection in *Rh. sanguineus* s.l. and *Rh. turanicus* s.s.; *E. canis* + *D. immitis* (4%) double co-infection in *Rh. sanguineus* s.l. and *Rh. turanicus* s.s. ticks; *D. immitis* + *Wolbachia* sp. (2%) and *E. canis* + *Ehrlichia* sp. (2%) double co-infection in *Rh. sanguineus* s.l. ticks, respectively. DNA of three V/TBPs was detected in five (10%) tick samples. Triple co-infection was observed for *E. canis* + *R. massiliae* + *R. raoultii* (4%) in *Rh. sanguineus* s.l. and *Rh. turanicus* s.s. ticks followed by *E. canis* + *D. immitis* + *Wolbachia* sp. (4%) in *Rh. sanguineus* s.l. and *R. massiliae* + *Rickettsia* sp. + *T. annulata* (2%) triple co-infection in *Hy. dromedarii*. The associations among the different tick species and patterns of infections, viz. single (*χ*^2^ = 11.890, *df* = 5, *P* = 0.014), double (*χ*^2^ = 11.11, *df* = 3, *P* = 0.011), and triple (*χ*^2^ = 8.26, *df* = 2, *P* = 0.017) were found statistically significant (*P* < 0.05) (Table 3).

**Table 3:** Single and mixed V/TBP infection in ixodid tick species collected from dogs across the study area

Ixodid tick species	Frequency of V/TBPs infection in ixodid tick species																							
	Single infection *n* (%)										Mixed (Co-infection) infection *n* (%)													
	Ec	Esp	Rm	Rr	Rsp	Ta	Di	Wsp	Total	Statistic	Double infection								Statistic	Triple infection				Statistic
											Ec + Esp	Ec + Rm	Rm + Rr	Rm + Rsp	Ec + Di	Di + Wsp	Total			Ec + Rm + Rr	Rm + Rsp + Ta	Ec + Di + Wsp	Total	
*Rh. sanguineus* s.l	1 (5)	1 (5)	3 (15)	2 (10)	1 (5)	0	1 (5)	1 (5)	10		1 (5)	2 (10)	1 (5)	1 (5)	1 (5)	1 (5)	7			1 (5)	0	2 (10)	3	
*Rh. turanicus* s.s	1 (5)	1 (5)	1 (5)	2 (10)	1 (5)	0	1 (5)	1 (5)	8	*χ*^*2*^ = 11.890	0	1 (5)	1 (5)	1 (5)	1 (5)	0	4		*χ*^*2*^ = 11.11	1 (5)	0	0	1	*χ*^*2*^ = 8.26
*Rh. haemaphysaloides*	1 (5)	1 (5)	1 (5)	0	1 (5)	1 (5)	0	0	5	*df* = 5	0	0	0	1 (5)	0	0	1		*df* = 3	0	0	0	0	*df* = 2
*Rh. microplus*	0	0	1 (5)	0	0	0	0	0	1	*P* = 0.014	0	0	0	0	0	0	0		*P* = 0.011	0	0	0	0	
*Hy. dromedarii*	0	0	4 (20)	0	1 (5)	1 (5)	0	0	6		0	0	0	1 (5)	0	0	1			0	1 (5)	0	1	*P* = 0.01
*Hy. excavatum*	0	0	1 (5)	0	0	1 (5)	0	0	2		0	0	0	0	0	0	0			0	0	0	0	
Total	32/50										13/50									5/50				

V/TBPs: vector/tick-borne pathogens; Ec: *Ehrlichia canis*, Esp: *Ehrlichia* sp.; Rm: *Rickettsia massiliae*; Rsp: *Rickettsia* sp.; Ta: *Theileria annulata*; Di: *Dirofilaria immitis*; Wsp: *Wolbachia* sp.

### Molecular features of V/TBPs gene sequences

The resultant partial nucleotide sequence size of the detected pathogen's genes was: *Ehrlichia canis 16S rRNA* = 281 bp, *Ehrlichia* sp. *16S rRNA* = 334 bp; *R. massiliae 16S rRNA*/*gltA* = 305/348 bps*, R. raoultii 16S rRNA*/*gltA* = 316/353 bps, *Rickettsia* sp. *16S rRNA*/*gltA* = 713/344 bps; *Wolbachia* sp. *16S rRNA* = 305 bp; *T. annulata 18S rRNA* = 980 bp and *D. immitis cox1* = 166 bp, respectively (Additional file 1: Table S1). The characterized gene sequences can be retrieved from NCBI GenBank with the following accession numbers (*E. canis 16S rRNA*: OP605546-OP605547; *Ehrlichia* sp. *16S rRNA*: ON926911-ON926912; *R. massiliae 16S rRNA*: ON909181-ON909182, *gltA*: ON952468-ON952469; *R. raoultii 16S rRNA*: ON909183-ON909184, *gltA*: ON952466-ON952467; *Rickettsia* sp. *16S rRNA*: ON909179-ON909180, *gltA*: ON952470-ON952471; *T. annulata 18S rRNA*: ON982513-ON982514; *D. immitis cox1:*OQ600716, OQ600720-OQ603322 OQ600728 OQ600729 OQ603256 OQ603257 OQ601560 OQ601563 OQ600765 OQ600766; *Wolbachia* sp. *16S rRNA*: OQ379166-OQ379169).

Online BLAST analysis of *16S rRNA* isolates of *E. canis* and *Ehrlichia* sp. shared 100% and 99–100% homology with the same isolates published from Asia, Europe, South Africa and the USA. Likewise, partial *16S rRNA*/*gltA* nucleotide isolates showed 98–100% sequence similarity with identical published isolates in NCBI GenBank from Asian, European and African countries. Similarly, partial *18S rRNA* isolates of *T. annulata*, partial *cox1* nucleotide sequences of *D. immitis* and partial *16S rRNA* amplicons of *Wolbachia* sp. shared up to 99.90%, 100% and 99.01–100% sequence similarity with the same species isolates reported from Asia, Europe and South America, respectively (Additional file 1: Tables S2 and S3).

### Phylogenetic profile of detected V/TPBs

**Fig. 3:**
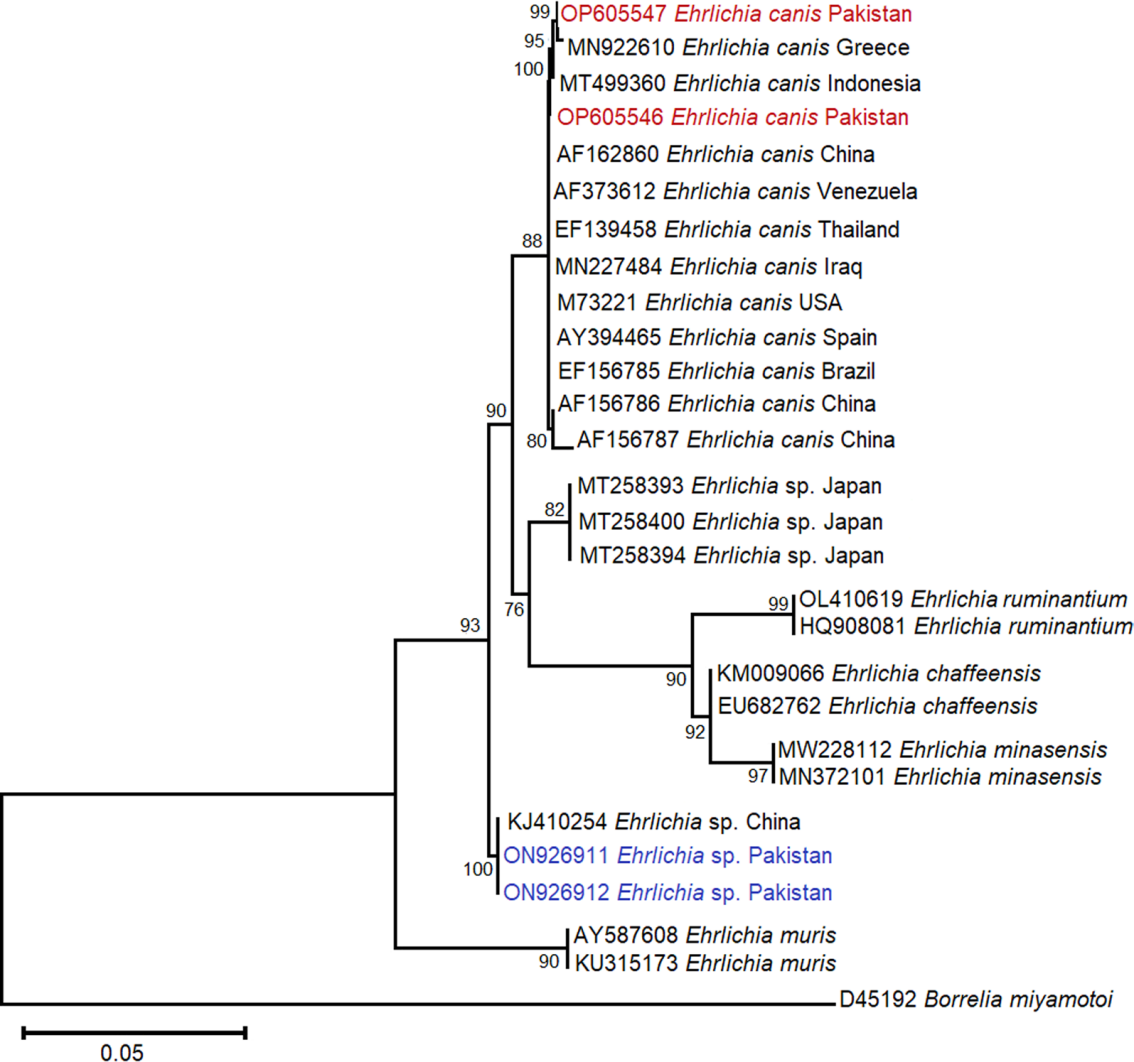
Phylogenetic analysis of *Ehrlichia canis* and *Ehrlichia* sp. isolated from ixodid ticks (this study: colored) with reference *Ehrlichia* sp. isolates (NCBI GenBank) based on partial *16S rRNA* gene sequences. The phylogram was constructed using the maximum likelihood algorithm based on the Kimura two-parameter model. The dataset was resampled 1000 times for bootstrap value generation. *Borrelia miyamotoi* (D45192) was used as the outgroup

The *16S rRNA*-based phylograms were computed to infer the evolutionary relatedness of the species belonging to genera *Ehrlichia*, *Rickettsia* and *Wolbachia* species. The detected rickettsiae *16S rRNA* isolate-based phylogenies were further supported by *gltA* isolate-based phylogram. Among the Ehrlichial microorganisms, *E. canis* specimens under this study clustered with the similar specimen on the *16S rRNA* phylogram published from Europe (Greece MN922610, Spain AY394465), Asia including the Far East (China AF162860, Indonesia MT499360, Iraq MN227484, Thailand EF139458), North America (USA M73221) and South America (Brazil EF195134, Venezuela AF373612) with bootstrap support of 88%. The *16S rRNA*-based ML phylogram showed that *E. canis* isolates from the present study clustered together with isolates reported from Greece and Indonesia as distinct sub-clades with bootstrap support of 95%, 99% and 100%, suggesting that *E. canis* isolates were past descendant to the same isolates reported from the aforementioned countries (Fig. 3). On the other hand, unidentified *Ehrlichia* sp. detected in the current study were closely grouped with identical isolates from China (KJ410254) with a maximum bootstrapping of 100% (Fig. 3).

**Fig. 4:**
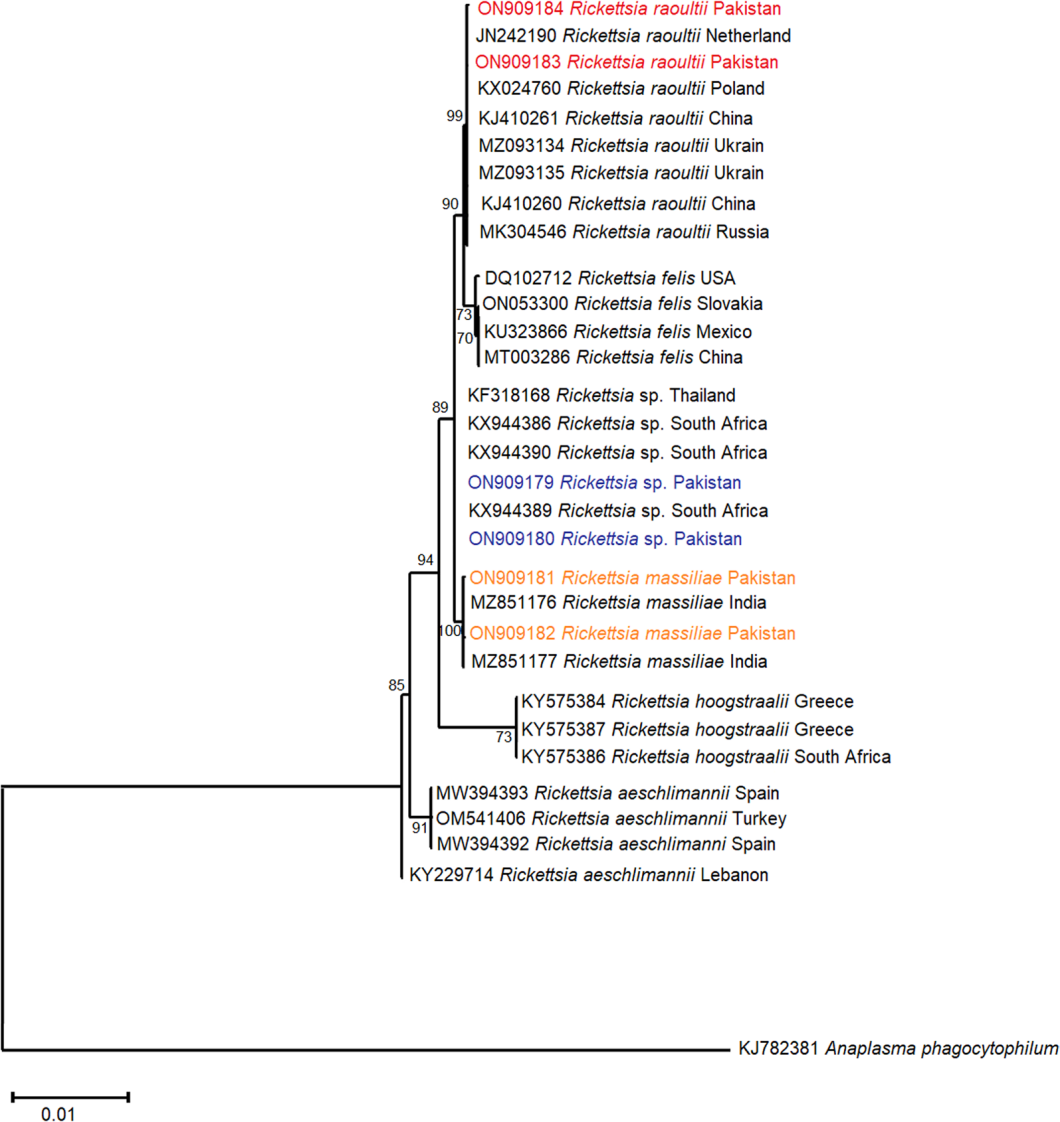
Phylogenetic analysis of *R. massiliae*, *Rickettsia raoultii* and *Rickettsia* sp. isolated from ixodid ticks (this study: colored) with reference *Rickettsia* sp. isolates (NCBI GenBank) based on partial *16S rRNA* gene sequences. The phylogram was constructed using the maximum likelihood algorithm based on the Kimura two-parameter model. The dataset was resampled 1000 times for bootstrap value generation. *Anaplasma phagocytophilum* (KJ782381) was used as the outgroup

**Fig. 5:**
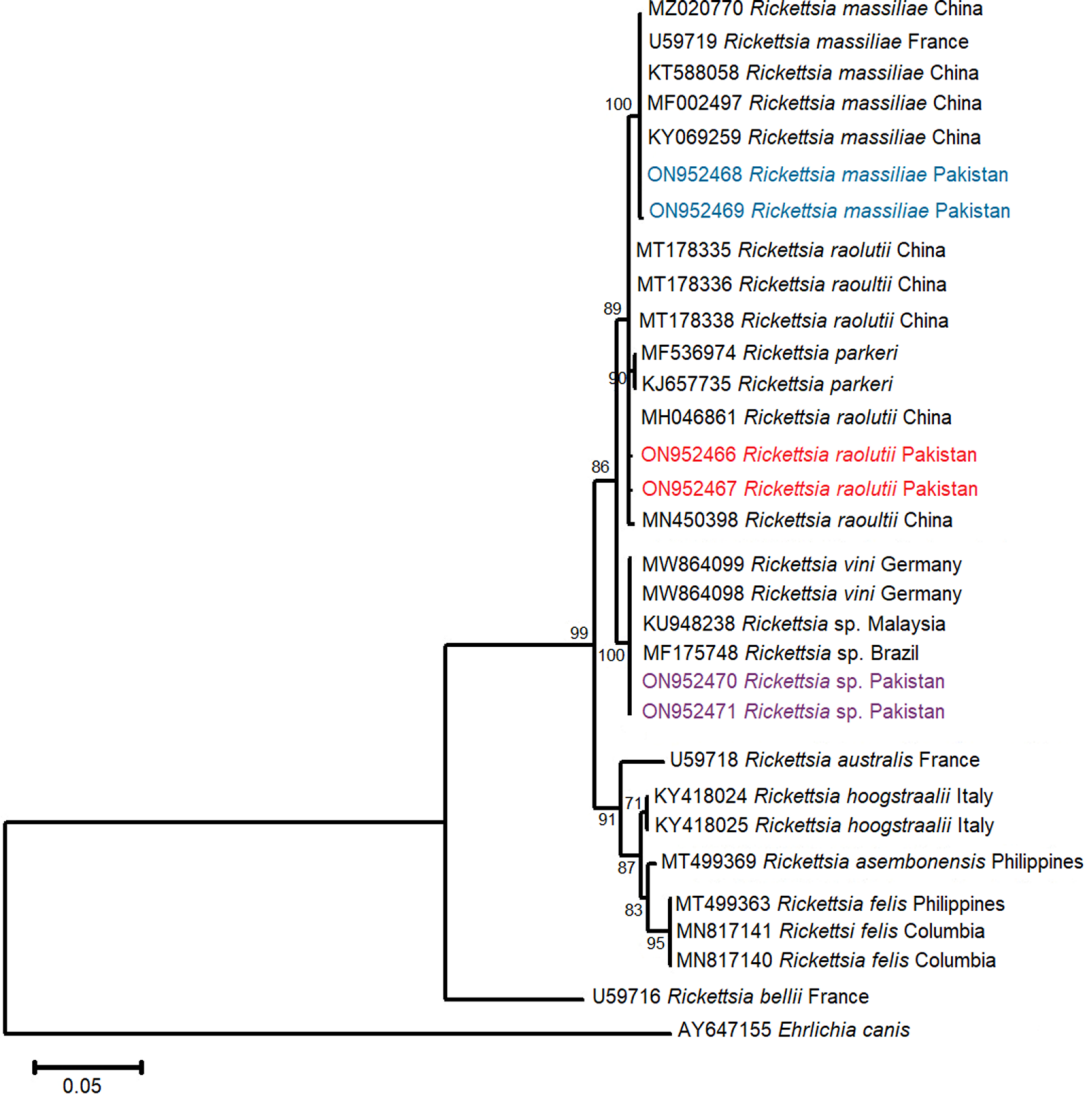
Phylogenetic analysis of *Rickettsia massiliae*, *R. raoultii,* and *Rickettsia* sp. isolated from ixodid ticks (this study: colored) with reference *Rickettsia* sp. isolates (NCBI GenBank) based on partial *gltA* gene sequences. The phylogram was constructed using the maximum likelihood algorithm based on the Tamura three-parameter model. The dataset was resampled 1000 times for bootstrap value generation. *Ehrlichia canis* (AY647155) was used as the outgroup

Among the Rickettsial bugs, zoonotic partial *R. massiliae 16S rRNA* nucleotide sequences shared an evolutionary relationship with similar isolates available in the NCBI GenBank repository from a neighboring Asian country (India MZ851176, MZ851177) with a maximum bootstrap value of 100%. Anthropozoonotic *R. raoultii 16S rRNA* specimens from the present study grouped with the same isolates reported from Asia (China KJ410260, Russia MK304546) and Europe (Netherlands JN242190, Ukraine MZ093134, Poland KX024760) with a bootstrap support of 99%. The unclassified *Rickettsia* sp. *16S rRNA* isolates from the present study showed evolutionary relatedness to the same pathogen’s sequences published from Asia (Thailand KF318168) and Africa (South Africa KX944386) with considerable bootstrap support of 89% (Fig. 4). The rickettsial *gltA* sequence-based phylogenetic tree showed that *R. massiliae gltA* isolates shared a phylogenetic relationship with identical specimens published from Asia (China KY069259, MF002497) and Europe (France U59719) with bootstrap support of 100%. Similarly, partial *R. raoultii gltA* nucleotide sequences shared evolutionary relatedness with the same gene sequences reported from a neighboring country (China MN046861, MN450398) with considerable branch support of 90%. However, the unclassified *Rickettsia* sp. *gltA* isolates clustered together with the identical isolates deposited in NCBI GenBank from Asia (Malaysia KU948238) and South America (Brazil MF175748) along with known *Rickettsia vini* isolates from Germany (MW864098 and MW864099) (Fig. 5).

**Fig. 6:**
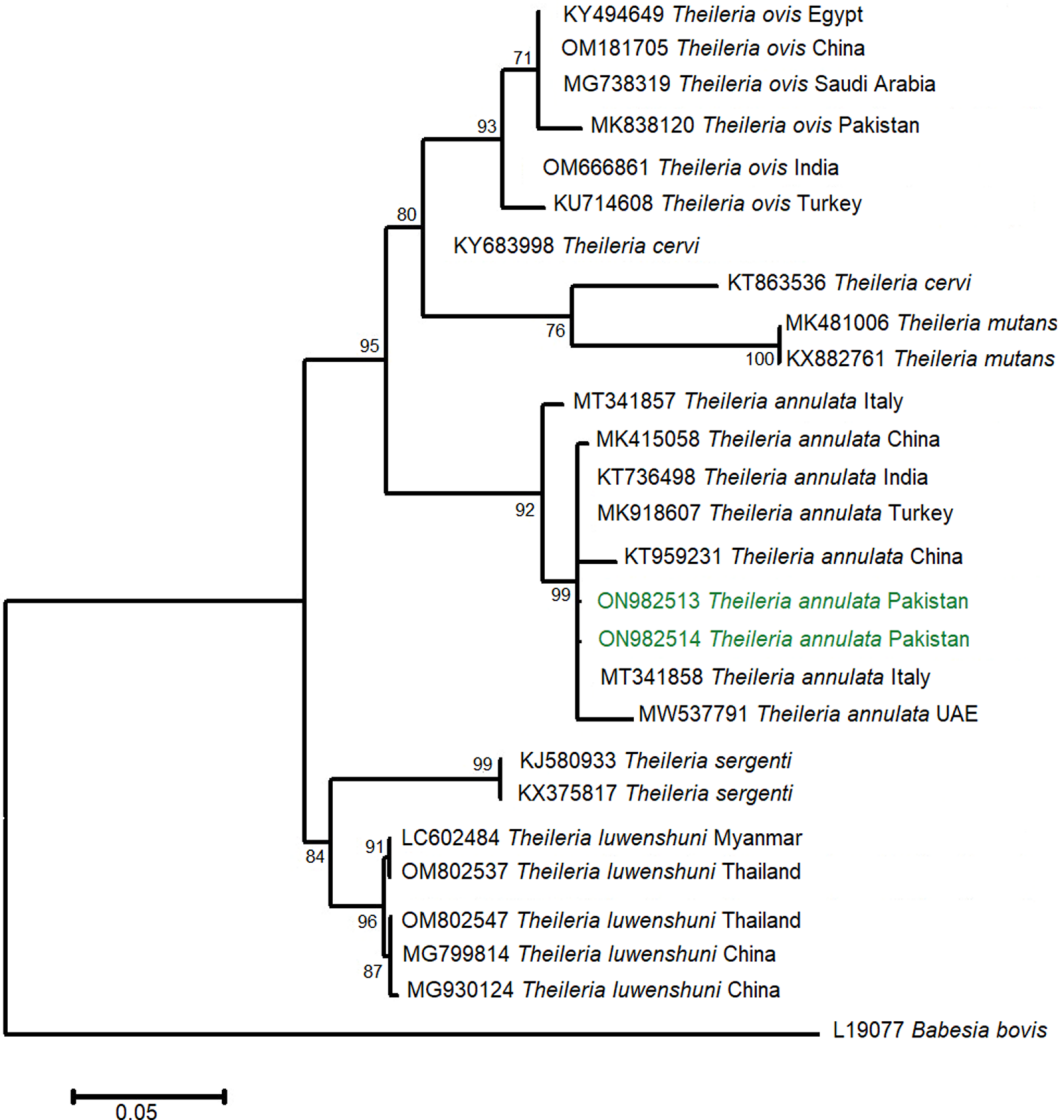
Phylogenetic analysis of *Theileria annulata* isolated from ixodid ticks (this study: colored) with reference *Theileria* sp. isolates (NCBI GenBank) based on partial *18S rRNA* gene sequences. The phylogram was constructed using the maximum likelihood algorithm based on the Kimura two-parameter model. The dataset was resampled 1000 times for bootstrap value generation. *Babesia bovis* (L19077) was used as the outgroup

The *T. annulata 18S rRNA* isolates from the present study shared a phylogenetic relationship with similar gene sequences published from Asia (China MK415058, KT959231; India T736498; UAE MW537791) and Europe (Turkey MK918607; Italy MT341858) with 99% bootstrap support (Fig. 6).

**Fig. 7:**
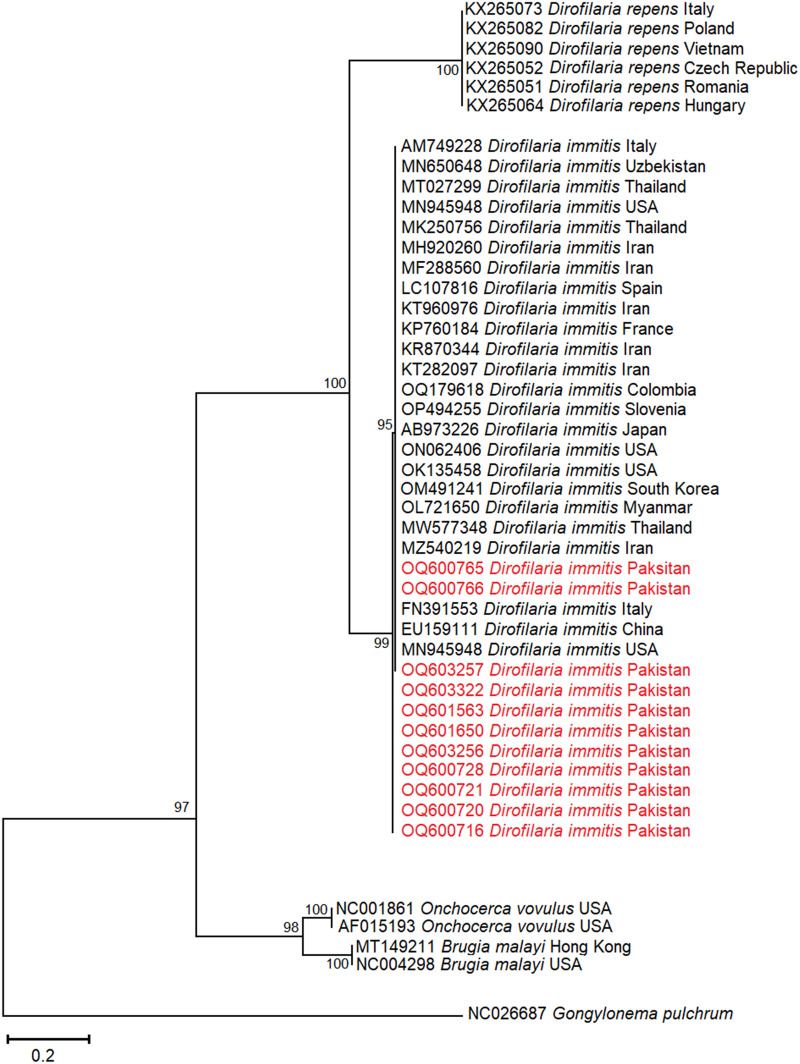
Phylogenetic analysis of *Dirofilaria immitis* isolated from ixodid ticks (this study: colored) with reference *Dirofilaria* sp. isolates (NCBI GenBank) based on partial *cox1* gene sequences. The phylogram was constructed using the maximum likelihood algorithm based on the Tamura three-parameter model. The dataset was resampled 1000 times for bootstrap value generation. *Gongylonema pulchrum* (NC026687) was used as the outgroup

The partial *D. immitis cox1* nucleotide sequences showed that the present study's dog heartworm samples clustered with the same species sequences separated from diverse host species (human, dog, cat and fox) across Asia (China EU159111; Iran MZ540219; Japan AB973226; Myanmar OL721650; Thailand MW577348; South Korea OM491241; Uzbekistan MN650648), Europe (France KP760184; Italy FN391553; Spain LC107816; Slovenia OP494255;), North America (USA MN945948, ON062406, MN945948) and South America (Colombia OQ179618) with maximum bootstrap support (100%). However, some *D. immitis* isolates detected during the present study clustered as separate sub-clades with considerable bootstrapping (95% and 99%) (Fig. 7).

**Fig. 8:**
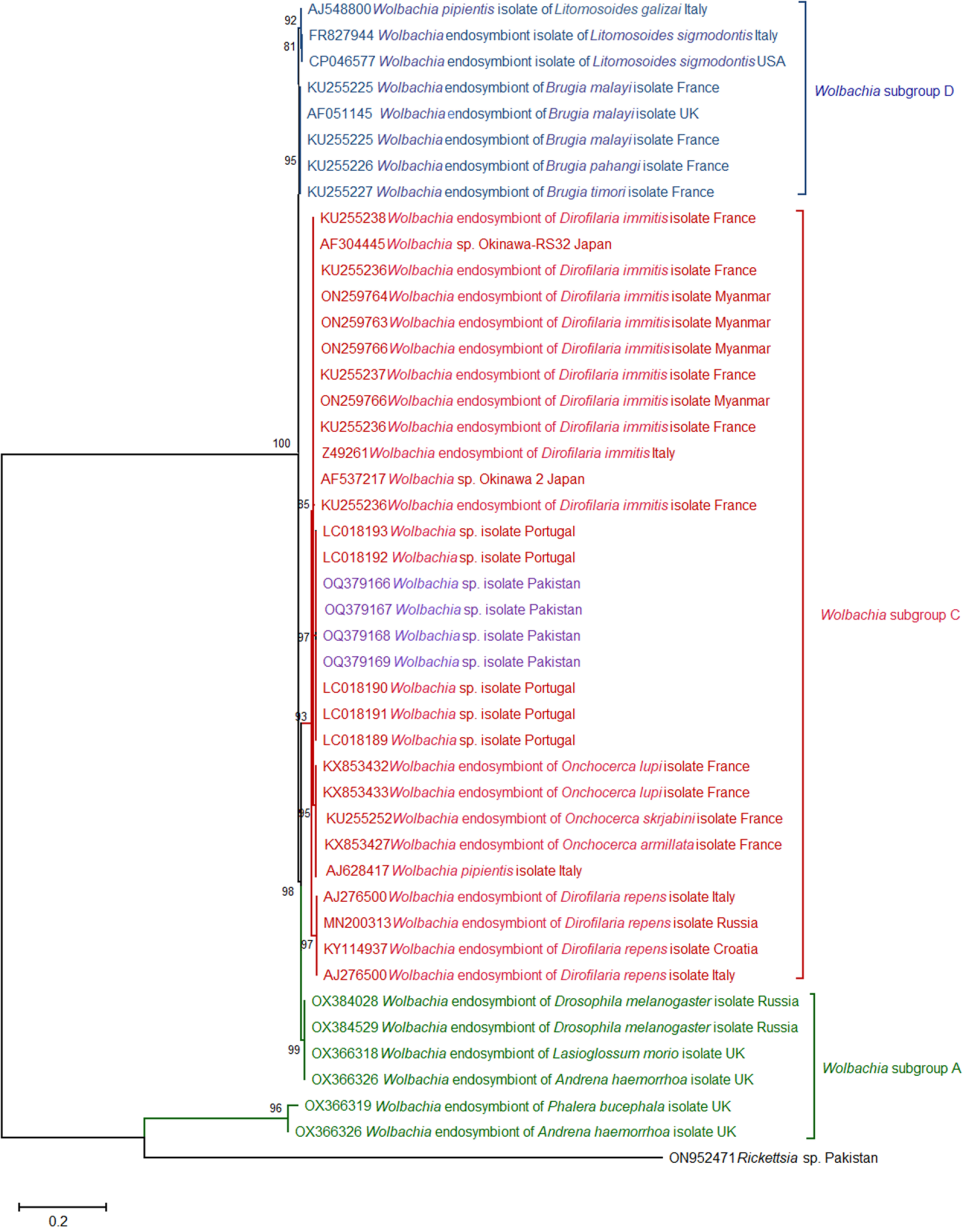
Phylogenetic analysis of *Wolbachia* sp. isolated from ixodid ticks (this study: purple colored) with reference *Wolbachia* sp. isolates (NCBI GenBank) based on partial *16S rRNA* gene sequences. The phylogram was constructed using the maximum likelihood algorithm based on the Tamura three-parameter model. The dataset was resampled 1000 times for bootstrap value generation. *Rickettsia sp.* (ON952471) was used as the outgroup

The partial *Wolbachia* sp. *16S rRNA* nucleotide sequences from the present study grouped with *Wolbachia* endosymbiont of filarial worm isolates (*Wolbachia* subclade C: infecting parasitic filarial nematodes) published in NCBI GenBank nucleotide repository from Asian and European countries with bootstrap statistical support of 93%. However, the present study's *Wolbachia* specimens shared a separate subclade within subgroup C along with unclassified *Wolbachia* sp. detected in dog blood samples in Portugal with considerable bootstrap statistic (97%). Further large-scale phylogenetic analysis of these unclassified *Wolbachia* sp. isolates is important to establish their accurate phylogenetic lineage (subgroup) (Fig. 8).

## Discussion

Climate change and global warming have facilitated disease vector spreading into new geographic locations and put the public and veterinary health workers at risk of contracting V/TBP infection in non-endemic areas [50]. Therefore, robust surveillance of disease vectors and associated pathogens in a particular geographic area is of utmost importance in devising appropriate vector and pathogen control interventions [15]. To the best of our knowledge, no comprehensive study has been conducted to explore the genetic diversity of V/TBPs in hard tick species infesting dog populations across Pakistan so far, with the exception of one mechanistic study (focused on the genetic diversity of *E. canis* in co-infected brown dog ticks) and two epidemiological investigations on pathogens detection in dog blood samples from Punjab province of Pakistan [51–53]. This is the first molecular report documenting a diverse array of zoonotic V/TBPs in ixodid ticks collected from dogs with implications for canine and public health from KP, Pakistan.

Vector-/tick-borne pathogens detected during the present study were grouped into five genera comprising eight species.

This study identified two species belonging to the genus *Ehrlichia* in ixodid ticks, viz. *E. canis* and *Ehrlichia* species. The zoonotic *E. canis* (causative agent of canine ehrlichiosis) was detected in *Rh. sanguineus* s.l., *Rh. turanicus* s.s. and *Rh. haemaphysaloides* ticks associated with dogs for the first time from KP, Pakistan. A significantly higher prevalence of the same pathogen was reported in dog blood samples and kennel tick *Rh. sanguineus* s.l. in Punjab province of Pakistan [51–53]. This fluctuation in the prevalence rate of *E. canis* across different agroecological zones of the country may be linked to the differences in sample size, diversity of infesting ticks and availability of competent tick vectors and host population that influence pathogen survival and in turn its prevalence rate. However, our findings are generally consistent with published literature that confirmed the presence of *E. canis* in the ixodid tick species and dog hosts from China, India, Korea, Philippines, Russia, Thailand, Portugal and Southeast Asia [3, 54–60]. The *16S rRNA* consensus sequences of *E. canis* shared homology and evolutionary history with identical isolates from Asia, Europe, North America and South America, suggesting that *E. canis*' identical genotype is circulating in different tick species globally without geographic restrictions [60–64]. Presence of *E. canis* in *Rh. sanguineus* s.l. ticks required special attention from veterinary health specialists in the study area as it is a competent vector and can transmit *E. canis* to a dog host [14]. So, proper vector control strategies should be adopted against this tick species.

The uncharacterized *Ehrlichia* sp. DNA was detected in four ixodid ticks during the present study. Our results regarding *Ehrlichia* sp. detection in hard ticks are parallel and support findings on *Ehrlichia* sp. detection in hard tick/dog blood samples from other agroecological zones of the country and elsewhere globally [55, 65–68]. *Ehrlichia* sp. phylogeny was established during the present study using *16S rRNA* nucleotide isolates. Similarly, multiple scientific reports inferred *Ehrlichia* sp. phylogeny successfully by using a *16S rRNA* phylogenetic marker [69–71]. These unclassified isolates need further large-scale phylogenetic studies to establish/evaluate their taxonomic position and pathogenic potential for human and animal.

The present study detected and characterized two species of SFG *Rickettsia*, namely *R. massiliae* (causative agent of febrile illness with skin rash, lymphadenopathy and inoculation eschar) [72] and *R. raoultii* (causative agent of SENLAT) [27], and one unclassified *Rickettsia* sp. in hard ticks.

The SFG *R. massiliae* was recovered from all reported tick species involved in this study. These findings are in accordance with published reports that confirmed *R. massiliae*'s presence in *Rh. sanguineus* s.l. collected from dogs across Punjab, Pakistan [52], and other multiple ixodid tick species infesting dogs worldwide [73–75]. Additionally, the presence of *R. massiliae* in *Rh. sanguineus* s.l. and *Rh. turanicus* s.s. ticks is predictive of increased transmission risk to dog owners as these tick species act as its competent vectors [76, 77]. The phylogenetic position of *R. massiliae* was successfully established based on *16S rRNA* and rickettsial *gltA* gene isolates. Similar approaches were adopted elsewhere to infer the evolutionary history of *R. massiliae* using the same phylogenetic markers [73, 74, 78, 79]. Our study is supportive of their findings regarding rickettsial species detection and phylogenetic studies.

The SFG *R. raoultii* was identified in *Rh. sanguineus* s.l. and *Rh. turanicus* s.s. ticks, which frequently infest dogs. *Rickettsia raoultii* in a small number of dog blood samples (*n* = 2) has been reported by a comparative study from Pakistan and Iran so far [53]. This research work is the initial report on *R. raoultii* detection and circulation in ixodid ticks associated with dogs in different agroecological zones of KP, Pakistan. This scientific report is parallel and validates published literature on *R. raoultii* detection and isolation from ticks infesting animals and humans [80–85]. The presence of *R. raoultii* in *Rh. sanguineus* s.l. and *Rh. turanicus* s.s. ticks required special attention of public and veterinary health experts as their vector competence for *R. raoultii* has been confirmed. The presence of *R. raoultii* in competent vectors intensifies the likelihood of its transmission to those who are in frequent contact with pet dogs [76, 77]. The *R. raoultii 16S rRNA* and *gltA* isolates shared phylogeny with identical globally reported isolates. A parallel/similar approach was adopted to investigate and infer the molecular phylogeny of *R. raoultii* elsewhere [80, 83, 84].

*Rickettsia* sp. was detected in four ixodid tick species during this study. Phylogenetic analysis clustered *Rickettsia* sp. isolates with unidentified *Rickettsia* sp. available in the NCBI GenBank Nucleotide database. Our findings follow global literature that reported unidentified *Rickettsia* sp. from several ixodid ticks associated with livestock including companion animals [60, 86, 87]. The molecular phylogeny of detected *Rickettsia* sp. was successfully inferred by using *16S rRNA* and *gltA* isolates. These findings support the published data elsewhere that had successfully inferred unclassified *Rickettsia* sp. phylogeny [60]. Further molecular work is needed to establish the taxonomic profile of unclassified *Rickettsia* sp. along with the prediction of its zoonotic potential for public and veterinary health.

No canine-associated *Theileria* sp. was detected in ixodid ticks analyzed by this study except *T. annulata* (causing bovine tropical theileriosis) in three species of hard ticks. Similarly, numerous studies reported the presence of *Theileria* sp. in dogs and hard ticks collected from them [87–90]. The phylogenetic profile of *Theileria* sp. was established by grouping with other *T. annulata* isolates on an *18S rRNA* phylogram. Our results support scientific reports that detected *T. annulata* and inferred its phylogeny based on the *18S rRNA* marker in ixodid ticks collected from diverse host species including dogs [89, 90].

The dog heartworm *D. immitis* (causative agent of canine cardiopulmonary dirofilariasis) was detected in two ixodid tick species. To our knowledge, *Dirofilaria immitis* detection in ixodid tick species by this study is the first to science, and no report has been published on *D. immitis* microfilariae detection in tick species so far, as this roundworm parasite is vectored by culicid mosquitos to canine hosts. These findings suggest that *D. immitis* microfilariae may have reached their dead-end host (i.e. the tick) during the feeding of infected ticks on dogs or that this parasite has extended its range of intermediate/paratenic hosts. To determine the role of kennel ticks in the dissemination of this parasite, their vector competence for *D. immitis* microfilariae should be investigated. Additionally, the presence of *D. immitis* in ixodid ticks indicates that the dog population in the study area is likely infected with this roundworm and that dog owners may be at risk of anthropozoonotic *D. immitis* infection if bitten by an infected tick. The *D. immitis* was successfully detected, and its phylogenetic position was determined using the *cox1* universal barcode. Our report is consistent with published global literature that detected and inferred the evolutionary relationship of *D. immitis* recovered from diverse canine hosts including dogs using the same gene isolates [91–95].

The endosymbiotic *Wolbachia* sp. DNA was detected in *Rh. sanguineus* s.l. and *Rh. turanicus* s.s. ticks for the first time from Pakistan. Our findings follow the globally published data that detected uncharacterized *Wolbachia* sp. or *Wolbachia* endosymbiont of *D. immitis* in heartworm-positive dog blood samples [96, 97]. Research work has demonstrated that *Wolbachia* endosymbiont facilitates *D. immitis* microfilariae development (embryogenesis and molting) in the definitive host population [36]. Thus, the presence of *Wolbachia* endosymbiont potentiates *D. immitis* microfilariae's survival to exploit canine hosts effectively. Phylogenetic analysis of *16S rRNA* isolates of *Wolbachia* sp. from the present study clustered with subclade C, which includes *Wolbachia* endosymbiont of filarial nematodes [33], and confirmed the close resemblance of these isolates with *Wolbachia* endosymbiont of *D. immitis*.

The co-existence of V/TBPs was found in molecularly screened hard tick species. Double and triple co-infections of pathogens were detected in 3/10 and 1/10 of the analyzed tick samples. The dog-associated ticks, i.e. *Rh. sanguineus* s.l. and *Rh. turanicus* s.s., were found positive for a maximum of V/TBP species and indicative of the high-level risk of pathogen transmission to humans and animals upon exposure to co-infected tick bite/infestation. A plethora of previously published data on vector or host-microbiome characterization revealed the co-occurrence of V/TBP and endosymbiont in ticks and canids [52, 73, 98, 99]. However, the composition and pattern (double/triple and so on) of co-infection varies regarding pathogen, host and vector species involved in time and space and is influenced by climatic factors, in particular landscape/agroecological zone. Additionally, levels of infection may differ among unfed, partially fed and completely engorged ticks [100]. To date, no human case has been reported involving co-infection of V/TBPs in Pakistan. The co-existence of V/TBPs in hard tick fauna across the study area is of public health interest as most of the pathogens detected by this study share common reservoir hosts and vector ticks, which assure co-infection transmission to dog owners.

## Conclusion

This scientific work is a comprehensive report on V/TBPs detection, their genetic diversity and prevalence patterns in ixodid ticks collected from dogs across KP, Pakistan. Five genera of V/TBPs were detected in molecularly screened hard ticks, i.e. *Ehrlichia, Rickettsia, Theileria*, *Dirofilaria* and *Wolbachia*. To our knowledge, detection of *D. immitis* in ixodid ticks is first to science while *E. canis* and *R. raoultii*'s presence in hard ticks is first from Pakistan. Phylogenetic analysis showed that V/TBP isolates shared homology and evolutionary histories with identical isolates published in the NCBI GenBank nucleotide repository from Old and New World countries. Among the reported pathogens, *E. canis, R. raoultii*, *R. massiliae,* and *D. immitis* were zoonotic V/TBPs of public and canine health concerns. The brown dog ticks *Rh. sanguineus* s.l. and *Rh. turanicus* s.s. carried a maximum of the detected pathogens, and for some of them *Rh. sanguineus* s.l. and/or *Rh. turanicus* s.s. is also a competent vector. During this study, there was evidence of the co-occurrence of various vector- and tick-borne pathogens. This raises the possibility of co-infections being transmitted to humans who have frequent contact with pet dogs. Physicians in Pakistan should be aware of the detection of V/TBPs in competent vector ticks and consider these pathogens in the diagnosis of patients with a history of tick bites from the study area. Moreover, it is recommended that further large-scale scientific investigations be conducted to explore the exclusive microbiome (pathobiome) of ixodid ticks associated with diverse host species in Pakistan. Such research would have significant implications for both public and veterinary health, including canid pets.

## Supplementary Information

**Additional file 1. Table S1**: Vector/tick-borne pathogen target gene amplicon size and nucleotide composition. **Table S2**: Vector/tick-borne pathogen target gene consensus sequences. **Table S3**: Vector/tick-borne pathogen target gene sequenceNCBI GenBank BLAST summary.

## Data Availability

The dataset and related information generated during the present study have been included in this manuscript in the best interest of the public and veterinary health.

## Abbreviations

SFG: Spotted fever group
TG: Typhus group
SENLAT: Scalp eschar and neck lymphadenopathy
PCR: Polymerase chain reaction
*16S** rRNA*: 16S ribosomal RNA gene
*18S** rRNA*: 18S ribosomal RNA gene
*gltA*: Rickettsial citrate synthase-encoding gene
*cox1*: Cytochrome c oxidases subunit 1 gene
KP: Khyber Pakhtunkhwa
V/TBPs: Vector/tick-borne pathogens
sp.: Species
