# Supplementary material for: Genetic diversity of vector-borne pathogens in ixodid ticks infesting dogs from Pakistan with notes on Ehrlichia canis, Rickettsia raoultii and Dirofilaria immitis detection
Source: Parasit Vectors. 2023 Jun 28;16:214. doi: 10.1186/s13071-023-05804-2 (PMC10303367; doi:10.1186/s13071-023-05804-2)
Supplement: Supplementary file 1 — Additional file 1. Table S1: Vector/tick-borne pathogen target gene amplicon size and nucleotide composition. Table S2: Vector/tick-borne pathogen target gene consensus sequences. Table S3: Vector/tick-borne pathogen target gene sequenceNCBI GenBank BLAST summary. [file 13071_2023_5804_MOESM1_ESM.docx]

**Table S1.** Vector/Tick-borne pathogens target genes amplicons size and nucleotide compositions.

| V/TBPs | Gene | Amplicon Size (bp) | Nucleotide (A+T/G+C) contents (%) | | | | | | |
| --- | --- | --- | --- | --- | --- | --- | --- | --- | --- |
|  |  |  | A (n) | T (n) | G (n) | C (n) | A+T (%) | G+C (%) | Total count (all bases) |
| Genus *Ehrlichia* | | | | | | | | | |
| *E. canis* | *16S rRNA* | 281 | 85 | 65 | 87 | 44 | 53.4 | 46.6 | 281 |
| *Ehrlichia sp.* | *16S rRNA* | 334 | 97 | 76 | 103 | 58 | 51.8 | 48.2 | 334 |
| Genus *Rickettsia* | | | | | | | | | |
| *R. massiliae* | *16S rRNA/ gltA* | 305/348 | 90/91 | 64/124 | 91/70 | 60/63 | 50.5/61.8 | 49.5/38.2 | 305/348 |
| *R. raoultii* | *16S rRNA/ gltA* | 316/353 | 87/95 | 67/127 | 95/69 | 65/62 | 49.4/62.9 | 50.6/37.1 | 316/353 |
| *Rickettsia* sp. | *16S rRNA/ gltA* | 713/344 | 202/94 | 158/125 | 217/65 | 136/60 | 50.5/63.7 | 49.5/36.3 | 713/344 |
| Genus *Theileria* | | | | | | | | | |
| *T. annulata* | *18S rRNA* | 980 | 251 | 296 | 259 | 174 | 55.8 | 44.2 | 980 |
| Genus *Dirofilaria* | | | | | | | | | |
| *D. immitis* | *Cox1* | 166 | 35 | 81 | 30 | 20 | 69.9 | 30.1 | 166 |
| Genus *Wolbachia* | | | | | | | | | |
| *Wolbachia* sp. | *16S rRNA* | 305 | 94 | 75 | 90 | 46 | 55.4 | 44.6 | 305 |

**Table S2.** Vector/Tick-borne pathogens target genes consensus sequences.

| V/TBPs | Target Gene | Target gene amplicon sequence |
| --- | --- | --- |
| Genus *Ehrlichia* | | |
| *E. canis* | *16S rRNA* | AAGCGTTGTTCGGAATTATTGGGCGTAAAGGGCACGTAGGTGGACTAGTAAGTTAAAAGTGAAATACCAAAGCTTAACTTTGGAGCGGCTTTTAATACTGCTAGACTAGAGGTCGAAAGAGGATAGCGGAATTCCTAGTGTAGAGGTGAAATTCGTAGATATTAGGAGGAACACCAGTGGCGAAGGCGGCTATCTGGTTCGATACTGACACTGAGGTGCGAAAGCGTGGGGAGCAAACAGGATTAGATACCCTGGTAGTCCACGCTGTAAACGATGAGTGC |
| *Ehrlichia sp.* | *16S rRNA* | TCCCGGCAAACTCCGTGCCAGCAGCCGCGGTAATACGGAGGGGGCAAGCGTTGTTCGGAATTATTGGGCGTAAAGGGCACGTAGGTGGACTAGTAAGTTAAAAGTGAAATACCAAAGCTTAACTTTGGAGCTGCTTTTAATACTGCTAGACTAGAGTTCGAAAGAGGATAGTGGAATTCCTAGTGTAGAGGTGAAATTCGTAGATATTAGGAGGAACACCAGTGGCGAAGGCGGCTATCTGGTTCGATACTGACACTGAGGTGCGAAAGCGTGGGGAGCAAACAGGATTAGATACCCTGGTAGTCCACGCTGTAAACGATGAGTGCTAAATGTG |
| Genus *Rickettsia* | | |
| *R. massiliae* | *16S rRNA/gltA* | AGTTAGTGGCAGACGGGTGAGTAACACGTGGGAATCTACCCATCAGTACGGAATAACTTTTAGAAATAAAAGCTAATACCGTATATTCTCTACGGAGGAAAGATTTATCGCTGATGGATGAGCCCGCGTCAGATTAGGTAGTTGGTGAAGTAATGGCTCACCAAGCCGACGATCTGTAGCTGGTCTGAGAGGATGATCAGCCACACTGGGACTGAGACACGGCCCAGACTCCTACGGGAGGCAGCAGTGGGGAATATTGGACAATGGGCGAAAGCCTGATCCAGCAATACCGAGTGGAGTGATGA/ATCTGCGGAAGCCGATTGCTTTACTTACGACCCGGGTTTTATGTCTACTGCTTCTTGTCAGTCTACTATCACCTATATAGACGGTGATAAAGGAATCTTGCGGCATCGAGGATATGATATTAAAGACTTAGCTGAGAAAAGTGATTTTTTAGAAGTGGCATATTTACTGATTTATGGGGAACTACCAAGTGGCGAGCAGTATAATAATTTCACTAAACAGGTTGCGCATCATTCATTAGTGAATGAAAGATTACACTATTTATTTCAAACCTTTTGTAGCTCTTCTCATCCTATGGCTATTATGCTTGCGGCTGTCGGTTCTCTTTCGGCATTTTATCCTGATTTATT |
| *R. raoultii* | *16S rRNA/gltA* | GGGGCTTGCTCCAATTAGTTAGTGGCAGACGGGTGAGTAACACGTGGGAATCTACCCATCAGTACGGAATAACTTTTAGAAATAAAAGCTAATACCGCATATTCTCTACGGAGGAAAGATTTATCGCTGATGGATGAGCCCGCGTCAGATTAGGTAGTTGGTGAGGTAATGGCTCACCAAGCCGACGATCTGTAGCTGGTCTGAGAGGATGATCAGCCACACTGGGACTGAGACACGGCCCAGACTCCTACGGGAGGCAGCAGTGGGGAATATTGGACAATGGGCGAAAGCCTGATCCAGCAATACCGAGTGAGTG/CCGATTGCTTTACTTACGACCCGGGTTTTATGTCTACTGCTTCTTGTCAGTCTACTATCACCTATATAGACGGTGATAAAGGAATCTTGCGGCATCGAGGATATGATATTAAAGACTTAGCTGAGAAAAGTGATTTTTTAGAAGTGGCATATTTACTGATTTATGGGGAACTACCAAGTGGCGAGCAGTATAATAATTTCACTAAACAGGTTGCTCATCATTCATTAGTGAATGAAAGATTACACTATTTATTTCAAACCTTTTGTAGCTCTTCTCATCCTATGGCTATTATGCTTGCGGCTGTCGGTTCTCTTTCGGCATTTTATCCTGATTTATTGAATTTTAAGGAAGCA |
| *Rickettsia* sp. | *16S rRNA/gltA* | GCTTAACACATGCAAGTCGAACGGACTAATTGGGGCTTGCTCCAATTAGTTAGTGGCAGACGGGTGAGTAACACGTGGGAATCTACCCATCAGTACGGAATAACTTTTAGAAATAAAAGCTAATACCGTATATTCTCTACGGAGGAAAGATTTATCGCTGATGGATGAGCCCGCGTCAGATTAGGTAGTTGGTGAGGTAATGGCTCACCAAGCCGACGATCTGTAGCTGGTCTGAGAGGATGATCAGCCACACTGGGACTGAGACACGGCCCAGACTCCTACGGGAGGCAGCAGTGGGGAATATTGGACAATGGGCGAAAGCCTGATCCAGCAATACCGAGTGAGTGATG  AAGGCCTTAGGGTTGTAAAGCTCTTTTAGCAAGGAAGATAATGACGTTACTTGCAGAAAAAGCCCCGGCTAACTCCGTGCCAGCAGCCGCGGTAAGACGGAGGGGGCTAGCGTTGTTCGGAATTACTGGGCGTAAAGAGTGCGTAGGCGGTTTAGTAAGTTGGAAGTGAAAGCCCGGGGCTTAACCTCGGAATTGCTTTCAAAACTACTAATCTAGAGTGTAGTAGGGGATGATGGAATTCCTAGTGTAGAGGTGAAATTCTTAGATATTAGGAGGAACACCGGTGGCGAAGGCGGTCATCTGGGCTACAACTGACGCTGATGCACGAAAGCGTGGGGAGCAAACAGGATTAGATACCCTGGT/CTTTACTTACGACCCGGGTTTTATGTCTACTGCTTCTTGTCAGTCTACTATCACTTATATAGACGGTGATAAAGGAATCTTGCGGCATCGAGGATATGACATTAAAGACTTAGCTGAGAAAAGTGATTTTTTAGAAGTGGCATATTTACTGATTTATGGGGAACTACCAAGCATCGAGCAGTATAATAATTTCACTAAACAGGTTGCTCATCATTCATTAGTGAATGAAAGATTACACTATTTATTTCAAACCTTTTGTAGCTCTTCTCATCCTATGGCTATTATGCTTGCGGCTGTCGGTTCTCTTTCGGCATTTTATCCTGATTTATTGAATTTTAAGGAAG |
| Genus *Theileria* | | |
| *T. annulata* | *18S rRNA* | GCAGCCGCGGTAATTCCAGCTCCAATAGCGTATATTAAAATTGTTGCAGTTAAAAAGCTCGTAGTTGAATTTCTGCTGCATTGCTTGTGTCCCTCTGGGGTCTGTGCATGTGGCTTTTTTCGGACGGAGTTTCTTTGTCTGAATGTTTACTTTGAGAAAATTAGAGTGCTCAAAGCAGGCTTTCGCCTTGAATAGTTTAGCATGGAATAATAAAGTAGGACTTTGGTTCTATTTTGTTGGTTTTAGGTACCAAAGTAATGGTTAATAGGAACAGTTGGGGGCATTCGTATTTAACTGTCAGAGGTGAAATTCTTAGATTTGTTAAAGACGAACTACTGCGAAAGCATTTGCCAAGGATGTTTTCATTAATCAAGAACGAAAGTTAGGGGATCGAAGACGATCAGATACCGTCGTAGTCCTAACCATAAACTATGCCGACTAGAGATTGGAGGTCGTCAGTTTTTACGACTCCTTCAGCACCTTGAGAGAAATCAAAGTCTTTGGGTTCTGGGGGGAGTATGGTCGCAAGGCTGAAACTTAAAGGAATTGACGGAAGGGCACCACCAGGCGTGGAGCCTGCGGCTTAATTTGACTCAACACGGGGAAACTCACCAGGTCCAGACAAAGGAAGGATTGACAGATTGATAGCTCTTTCTTGATTCTTTGGGTGGTGGTGCATGGCCGTTCTTAGTTGGTGGAGTGATTTGTCTGGTTAATTCCGTTAACGAACGAGACCTTAACCTGCTAAATAGGGTACGGGAATAAGTTTCTACTGTCCCGTTATCGCTTCTTAGAGGGACTTTGCGGTTATAAATCGCAAGGAAGTTTAAGGCAATAACAGGTCTGTGATGCCCTTAGATGTCCTGGGCTGCACGCGCGCTACACTGATGCGTTCATCGAGTTTATCCTTGGCCGAGAGGCCTGGGTAATCTTTAGTACGCATCGTGATGGGGATCGATTATTGCAATTGTTAACGTGA |
| Genus *Dirofilaria* | | |
| *D. immitis* | *cox1* | GATTTTAGGTCTTCATACTGTTGGTATTGGTTCTTTATTAGGTGCTATTAATTTTATGGTTACTGTTCAGAATATACGTTCTACTGCTGTAACTTTAGATCAGATTAGTATGTTTGTTTGAACTTCTTATTTAACTTCTTTTTTATTGGTATTGTCAGTGCCTGTA |
| Genus *Wolbachia* | | |
| *Wolbachia* sp. | *16S rRNA* | GCCGCGGTATACGGAGAGGGCTAGCGTTATTCGGAATTATTGGGCGTAAAGAGCACGTAGGCTGGTTAGTAAGTTAAAAGTGAAATTCCAAAGCTTAACTTTGGAATTGCTTTTAAAACTGCTGATCTAGAGATTGAAAGAGGATAGAGGAATTCCTAGTGTAGAGGTGAAATTCGTAAATATTAGGAGGAACACCAGTGGCGAAGGCGTCTATCTGGTTCAAATCTGACGCTGAGGTGCGAAAGCGTGGGGAGCAAACAGGATTAGATACCCTGGTAGTCCACGCTGTAAACGATGAGTGCTAA |

**Table S3.** Vector/Tick-borne pathogens target genes sequences (query) NCBI GenBank BLAST summary.

| V/TBPs from Dogs across Khyber Pakhtunkhwa Pakistan (Present study) | | | NCBI GenBank Repository Published Report  (Global Record) | | |
| --- | --- | --- | --- | --- | --- |
| V/TBPs species | Desired gene | Query Sequences  (Accession No.) | Reference/Subject Sequences  (Accession No.) | BLAST  Similarity (%) | Country of Origin |
| *E. canis* | *16S rRNA* | OP605546, OP605547 | MT499360, MN922610, MN227484 | 100 | China, Thailand  Greece, Iraq |
| *Ehrlichia sp.* | *16S rRNA* | ON926911, ON926912 | KJ410254, JX402603, MH25019, MZ351091, AF497581 | 99.40-100 | China, Pakistan, Eswatini, Vietnam |
| *R. massiliae* | *16S rRNA/gltA* | ON909181, ON909182/  ON952468, ON952469 | MZ851177, KX944390, GQ144453  /MF002497, OM475669, OK205221 | 99.34-100/100 | India, South Africa, Spain/ China, Spain |
| *R. raoultii* | *16S rRNA/gltA* | ON909183, ON909184/ ON952466, ON952467 | MK304546, KX024760, KJ410260  /MT178338, MN450401, MH046861 | 100/100 | Russia, Poland, China/ China |
| *Rickettsia* sp. | *16S rRNA/gltA* | ON909179, ON909180/ ON952470, ON952471 | KF318168, KX944377, MT434770 /KJ769650, KU948238, KX591657 | 100/98.55-100 | Thailand, South Africa, Taiwan/ Malaysia, China |
| *T. annulata* | *18S rRNA* | ON982513, ON982514 | MT341858, KT736498, OQ120433, KF429800 | 99.90 | Italy, India, Turkey, Iran |
| *D. immitis* | *cox1* | OQ600716, OQ600721, OQ603322, OQ600729, OQ601560, OQ601563 | KC985239, MN650648, MT230089, KY247119, LC107816, KT282097 | 100 | Iran, Slovakia, Uzbekistan, France, Turkey, Spain |
| *Wolbachia* sp. | *16S rRNA* | OQ379166- OQ379169 | LC018189, KU255236, ON259766 | 99.01-100 | Portugal, France, Myanmar, |
